# Hepatitis B virus promotes β-catenin-signalling and disassembly of adherens junctions in a Src kinase dependent fashion

**DOI:** 10.18632/oncotarget.26103

**Published:** 2018-09-21

**Authors:** Gesa von Olshausen, Maria Quasdorff, Romina Bester, Silke Arzberger, Chunkyu Ko, Maarten van de Klundert, Ke Zhang, Margarete Odenthal, Marc Ringelhan, Carien M. Niessen, Ulrike Protzer

**Affiliations:** ^1^ Department of Internal Medicine I, University Hospital rechts der Isar, Technical University of Munich, Munich, Germany; ^2^ Molecular Infectiology, Institute for Medical Micro biology, Immunology and Hygiene, University Hospital Cologne, Cologne, Germany; ^3^ Department of Gastroenterology and Hepatology, University Hospital Cologne, Cologne, Germany; ^4^ Institute of Virology, Technical University of Munich/Helmholtz Zentrum München, Munich, Germany; ^5^ Institute of Pathology, University Hospital Cologne, Cologne, Germany; ^6^ Center for Molecular Medicine (CMMC), University of Cologne, Cologne, Germany; ^7^ Department of Internal Medicine II, University Hospital rechts der Isar, Technical University of Munich, Munich, Germany; ^8^ Department of Dermatology, University Hospital of Cologne, Cologne, Germany; ^9^ Cologne Excellence Cluster on Cellular Stress Responses in Aging-Associated Diseases (CECAD), University of Cologne, Cologne, Germany; ^10^ German Center for Infection Research (DZIF), Munich Partner Site, Munich, Germany

**Keywords:** hepatitis B virus (HBV), hepatocellular carcinoma (HCC), β-catenin, Src kinase, E-cadherin

## Abstract

Hepatitis B virus (HBV) infection is a prominent cause of hepatocellular carcinoma (HCC) but the underlying molecular mechanisms are complex and multiple pathways have been proposed such as the activation of the Wnt−/β-catenin-signalling and dysregulation of E-cadherin/β-catenin adherens junctions. This study aimed to identify mechanisms of how HBV infection and replication as well as HBV X protein (HBx) gene expression in the context of an HBV genome influence Wnt−/β-catenin-signalling and formation of adherens junctions and to which extent HBx contributes to this.

Regulation of E-cadherin/β-catenin junctions and β-catenin-signalling as well as the role of HBx were investigated using constructs transiently or stably inducing replication of HBV+/−HBx in hepatoma cell lines. In addition, HCC and adjacent non-tumorous tissue samples from HBV-infected HCC patients and drug interference in HBV-infected cells were studied.

Although HBV did not alter overall expression levels of E-cadherin or β-catenin, it diminished their cell surface localization resulting in nuclear translocation of β-catenin and activation of its target genes. In addition, HBV gene expression increased the amount of phosphorylated c-Src kinase. Treatment with Src kinase inhibitor Dasatinib reduced HBV replication, prevented adherens junction disassembly and reduced β-catenin-signalling, while Sorafenib only did so in cells with mutated β-catenin. Interestingly, none of the HBV induced alterations required HBx.

Thus, HBV stimulated β-catenin-signalling and induced disassembly of adherens junctions independently of HBx through Src kinase activation. These pathways may contribute to hepatocellular carcinogenesis and seem to be more efficiently inhibited by Dasatinib than by Sorafenib.

## INTRODUCTION

With over 800 000 deaths in 2015, liver cancer represents the fourth leading cause of cancer death in the world [1]. Hepatocellular carcinoma (HCC) is the dominant histologic type of liver cancer in most countries accounting for approximately 80% of total cases [2]. Hepatitis B virus (HBV) is one of the most common chronic infections worldwide with an estimated 257 million chronically infected subjects (WHO 2017). It is the leading cause for hepatocellular carcinoma and accounted for > 880.000 deaths (WHO 2017) and at least 265 000 liver cancer deaths in 2015 [1]. A plethora of different mechanisms through which HBV contributes to HCC initiation and progression have been described (summarized in [3]) and are subject of current investigations.

Hepatitis B virus (HBV) is the prototypic member of the *hepadnaviridae*, a family of small enveloped DNA-containing viruses. Because of their replication via reverse transcription of a pregenomic RNA (pgRNA), they are classified as pararetroviruses. HBV contains four open reading frames encoding the structural capsid, envelope proteins, the viral polymerase that serves as reverse transcriptase and the non-structural HBV X protein (HBx). Infection with HBV induces Src tyrosine kinase activity to promote its replication on the transcriptional and post-transcriptional level [4, 5].

The role of HBV in tumor formation is complex and likely involves both direct and indirect mechanisms [3, 6, 7]. Integration of HBV-DNA into the host genome may cause mutations and/or activation of diverse cancer-related genes, which subsequently drive clonal hepatocyte expansion at an early stage of HCC development [8]. Moreover, chronic liver inflammation and hepatic regeneration induced by cellular immune responses may also favour the accumulation of genetic alterations in infected hepatocytes [9]. Over the last years a growing body of evidence suggests that in addition to these mentioned well-established mechanisms pleiotropic effects of viral HBV proteins also contribute to HCC initiation and/or progression [10]. In this context, the HBV-encoded envelope and HBx proteins are reported to play an important role via distinct and non-overlapping pathways [3]. PreS/S mutants of HBV large surface antigens are suggested to induce endoplasmic reticulum stress via an unfolded protein response thereby leading to oxidative stress-induced DNA damage/mutation and activation of cell proliferation-related signals [11, 12]. In addition, numerous functions have been attributed to HBx: e.g. regulation of cytoplasmic calcium levels [13, 14], disruption of adherens junctions [15], induction of a migratory phenotype [16], activation of β-catenin-signalling [17] and recruiting the Smc5/6 complex for ubiquitination inducing its proteasomal degradation and thus influencing HBV gene expression [18]. HBx induced Smc5/6 degradation, however, may also influence cell division and DNA repair [3].

The canonical Wnt-signalling pathway determines cell fate in many tissues and organs [19, 20], with β-catenin as the key downstream signal transducer. Wnt binding to its receptor results in stabilization of cytosolic β-catenin and subsequent translocation to the nucleus where it interacts with DNA-binding proteins of the Tcf/LEF (T cell factor/lymphocyte enhancer factor) family [21, 22] to transcriptionally activate downstream targets, such as e.g. c-myc and glutamine synthase (GS) [23–25]. Dysregulation of Wnt/β-catenin-signalling is observed in a number of cancers, including HCC [26–28]. In mouse models, it has been shown that increased β-catenin-signalling directly contributes to HCC progression [29]. Next to its role in signalling, β-catenin also binds to the cytoplasmic domain of the cell adhesion protein E-cadherin and interacts with the actin binding protein α-catenin providing the E-cadherin/β-catenin complex with a dynamic link to the cytoskeleton. This cadherin/catenin complex forms the molecular backbone of the intercellular adherens junctions [30].

Loss of E-cadherin is a characteristic feature of epithelial to mesenchymal transition (EMT) and observed in a broad spectrum of human carcinomas [31–33]. This EMT induced E-cadherin loss is often accompanied by β-catenin nuclear translocation and signalling activation. In addition, it was shown that E-cadherin loss directly contributes not only to tumor invasion but also to tumor initiation [34]. Changes in E-cadherin expression or localization are also observed in human HCCs [35, 36] and in HCC mouse models [37]. However, in some HCC patients the frequently observed activation of β-catenin seemed to be independent of E-cadherin loss, suggesting additional mechanisms for β-catenin activation [35].

In the present study we investigated whether and how HBV stimulates β-catenin-signalling and disassembly of adherens junctions. We describe that both require c-Src kinase activation but not the viral transactivator HBx. Furthermore, we demonstrate that inhibition of Src kinase activity by Dasatinib restored adherens junctions, reduced β-catenin-signalling and repressed HBV replication.

## RESULTS

### HBV does not alter protein levels of E-cadherin/β-catenin

**Figure 1:**
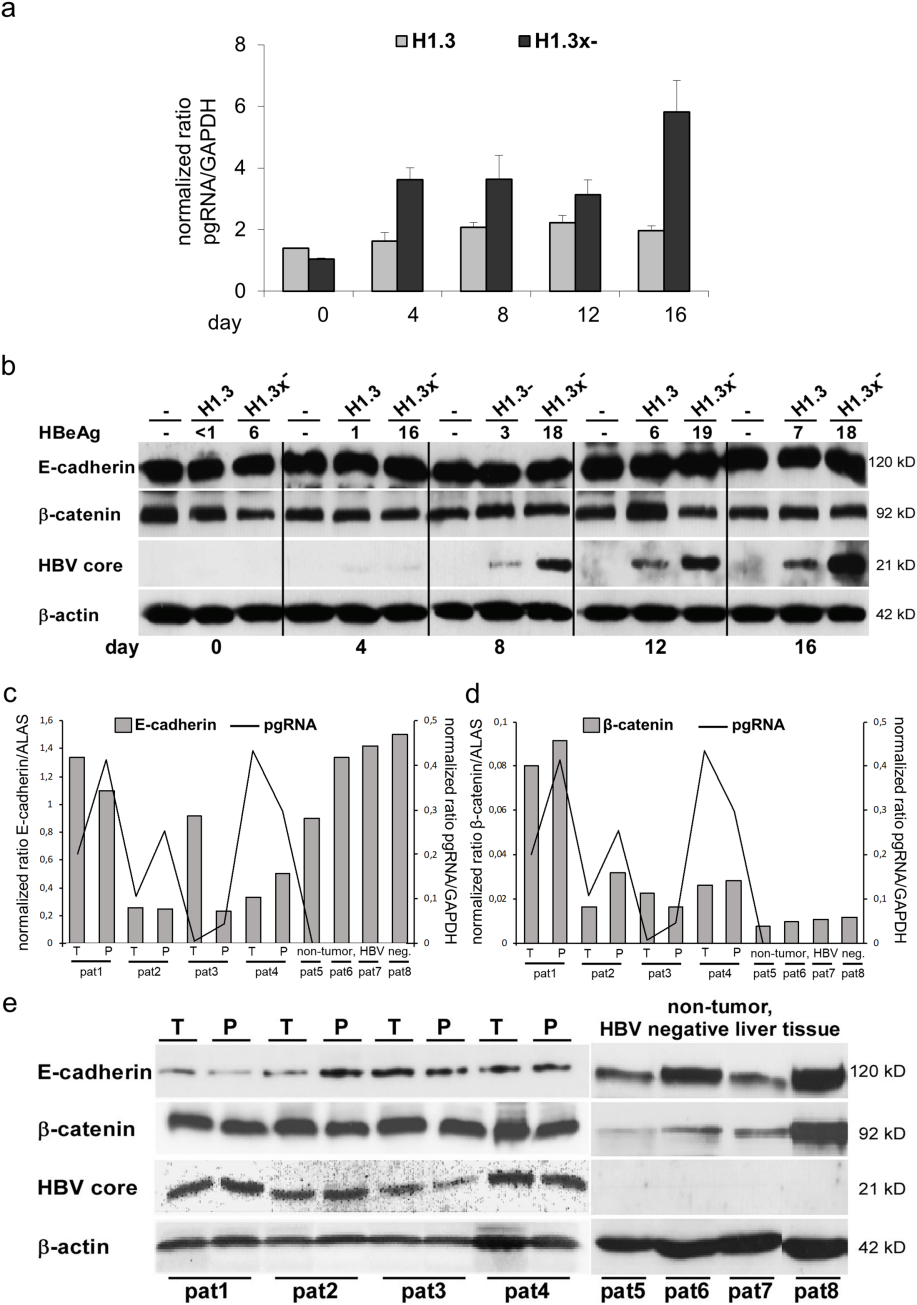
Total protein levels of E-cadherin/β-catenin in HBV-replicating hepatoma cells and mRNA expression/total protein levels of E-cadherin/β-catenin in human liver tissue (HBV-infected and HBV-negative) **(a)** HBV pgRNA (quantified by real-time PCR) in stably transfected HBV-producing HepG2 cell lines (H1.3 and H1.3x^–^) in a time course experiment. Day 0 was defined as day when cells were 80-90% confluent (here: 2 days post plating). mRNA and proteins were extracted at day 0, 4, 8, 12 and 16. Normalized expression ratios relative to housekeeping gene GAPDH are given. The assays were performed in independent triplicates, means + SD are shown. **(b)** Western blot analysis of E-cadherin, β-catenin and HBV core protein (same time course experiment) in parental (−) and stably transfected HBV-producing HepG2 cells (H1.3 and H1.3x^–^). HBeAg levels in cell culture supernatants are given in ng ml-1. β-actin was stained as loading control. **(c)** E-cadherin mRNA and **(d)** β-catenin mRNA as well as HBV pgRNA were quantified by real-time PCR in liver tissue of paired tumor (T)/peritumur (P) tissue samples of four HBV-infected human livers with HCC (pat1-pat4) and four non-tumor, HBV-negative livers (pat5-pat8). Normalized expression ratios relative to house keeping gene ALAS and GAPDH are given. **(e)** Western blot analysis of E-cadherin, β-catenin and HBV core protein of the same liver tissue lysates of paired tumour (T)/peritumour (P) tissue samples of four HBV-infected human livers with HCC (pat1-pat4) and four non-tumor, HBV-negative livers (pat5-pat8). β-actin was stained as loading control.

To examine whether HBV replication and expression of HBx under its endogenous promoter influence E-cadherin or β-catenin protein levels, we performed qPCR and Western blot analysis in HepG2 cell lines stably transfected with replication competent HBV overlength genome H1.3 or H1.3x^−^. Under conditions that have been shown to promote differentiation [38] such as plating onto a collagen matrix and medium containing dexamethasone, DMSO and low FCS, cells were allowed to differentiate for different times. In both cell lines, pgRNA (Figure 1a) and HBV replication levels (data not shown) increased over time during differentiation as previously described [39]. Western blot analysis revealed no change in either E-cadherin or β-catenin levels whereas HBV core protein and HBeAg levels steadily increased (Figure 1b). Investigating human tumor and peritumor liver tissue from individuals with and without HBV infection, numeric differences at E-cadherin (Figure 1c) and β-catenin (Figure 1d) mRNA expression levels could be detected. Nevertheless, there was no clear correlation with pgRNA in HBV infected patients (Figure 1c and 1d, left panel). In HBV negative patients, there was a trend to higher E-cadherin and lower β-catenin mRNA expression levels (Figure 1c and 1d, right panel). However, according to the results of the hepatoma cell lines' western blot, neither a difference in E-cadherin or β-catenin expression nor a correlation with HBV core expression levels could be detected when peritumor and tumor liver tissue from individuals with HBV infection were analyzed (Figure 1e, left panel). Moreover, no obvious difference between HBV-positive (Figure 1e, left panel) and HBV-negative patients was observed (Figure 1e, right panel). In fact, the most striking observation was the high inter-individual heterogeneity of E-cadherin and β-catenin protein levels. These data indicate that HBV does not directly regulate either E-cadherin or β-catenin expression levels, regardless of HBx expression.

### HBV alters E-cadherin/β-catenin localization

**Figure 2:**
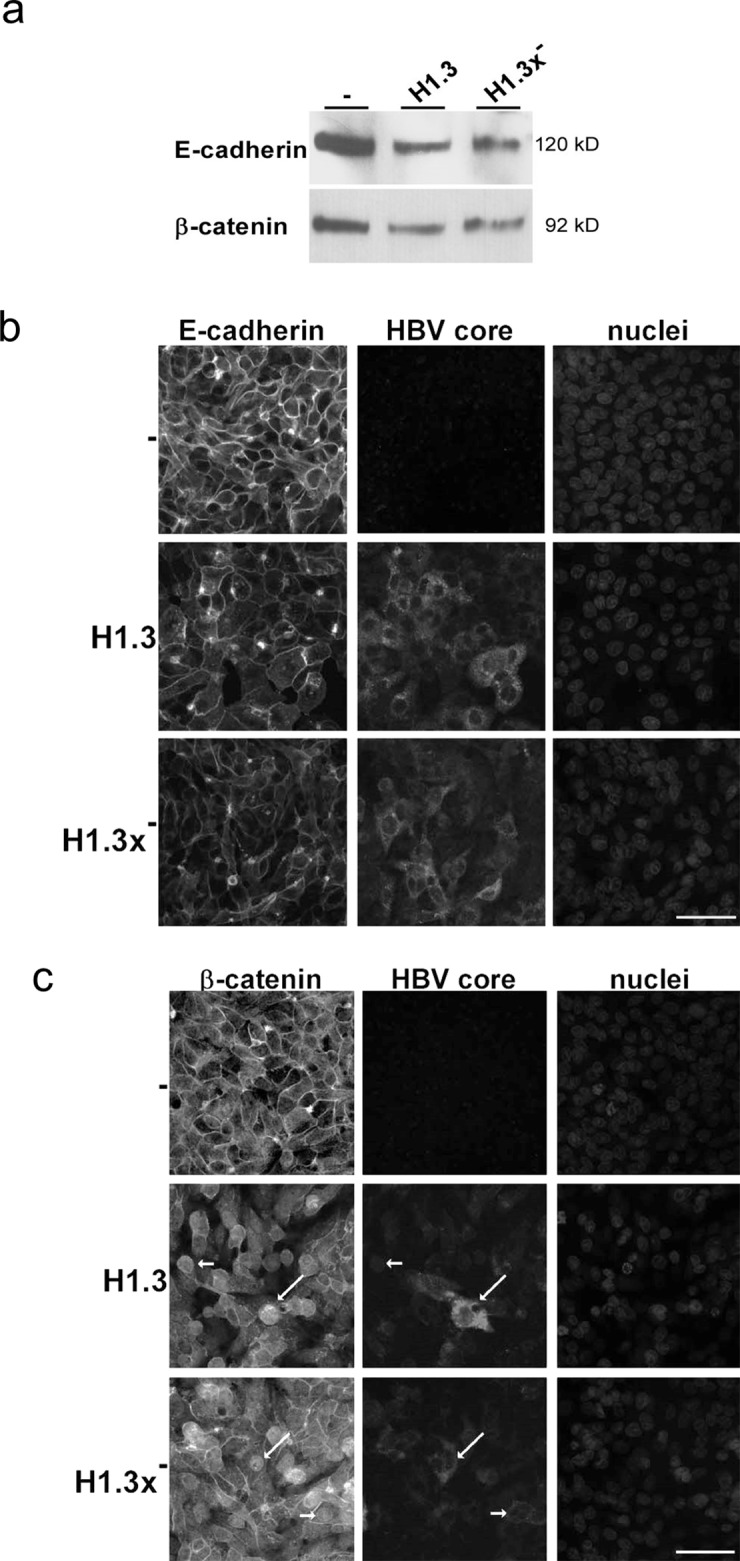
Distribution of E-cadherin/β-catenin in HBV-replicating hepatoma cells **(a)** Membrane fractions (isolated via a cell surface biotinylation assay) of E-cadherin and β-catenin in HBV-producing (H1.3 + H1.3x^−^) compared to parental (−) HepG2 cells were analyzed by Western blot. Immunoflourescence staining of **(b)** E-cadherin and **(c)** β-catenin (nuclear translocation indicated by arrows) in parental (−) and HBV-producing (H1.3 + H1.3x^−^) HepG2 cells. Co-staining of HBV core protein (indicated by arrows) and nuclei. Scale bars indicate 40μm.

Since HBV did not change overall E-cadherin or β-catenin protein levels we next investigated whether it affects E-cadherin/β-catenin localization. Cell surface biotinylation assays revealed diminished surface levels of E-cadherin and β-catenin in both HepG2 cells replicating HBV wildtype (H1.3) and x-deficient HBV (H1.3x^−^) compared to the parental control cell line (Figure 2a). To examine the subcellular localization of E-cadherin/β-catenin, immunofluorescence analysis was performed. Less E-cadherin was located to sites of cell-cell contacts in HepG2-H1.3 and -H1.3x^−^ cells when compared to control cells (Figure 2b). Instead, a punctuated pattern was observed in the cytoplasm, suggesting that E-cadherin was internalized or not efficiently transported back to the cell surface upon HBV replication. Similarly, an overall decrease in cell surface localization of β-catenin was observed in cells replicating HBV accompanied by a strong increase in both cytosolic and nuclear staining of β-catenin (Figure 2c). Interestingly, neither loss of cell surface localization of E-cadherin/β-catenin nor β-catenin translocation did depend on the presence of HBx (Figure 2a+2b+2c). Hence, these results demonstrate that HBV results in nuclear β-catenin translocation and reduces adherens junctions independently of HBx expression.

### HBV enhances β-catenin-signalling

**Figure 3:**
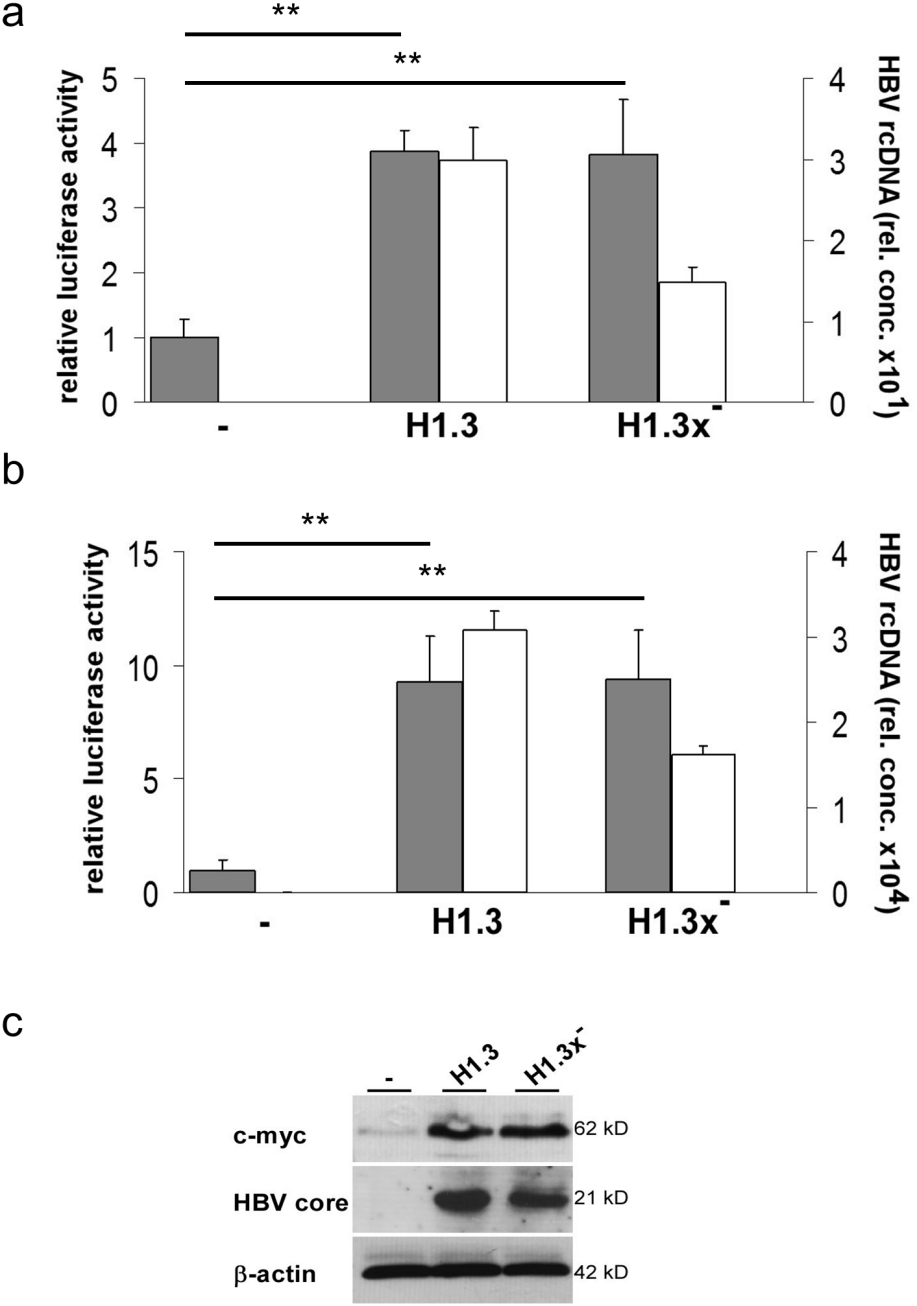
Tcf/LEF dependent β-catenin-signalling in HBV-replicating hepatoma cells Relative luciferase activity indicates the level of Tcf/LEF dependent β-catenin-signalling (grey bars) in parental (−) or H1.3x or H1.3x^−^ **(a)** replicating HepG2 and **(b)** transfected Huh7 cells. Ratios in parental cells were set to 1. Active viral replication was determined by measuring HBV rcDNA (white bars) levels in supernatants of HBV-producing cells. The assays were performed in independent triplicates, means + SD are shown. ^**^ p<0.01 vs parental cells, t-test. **(c)** c-myc and HBV core protein levels were subjected to Western blot analysis in parental (−) compared to HBV-replicating (H1.3 + H1.3x^−^) HepG2 cells.

The strong increase in cytosolic and nuclear β-catenin levels indicated that HBV may activate β-catenin-signalling. Tcf/LEF dependent luciferase reporter assays revealed a 3.8-fold enhanced β-catenin-signalling in both HepG2-H1.3 and HepG2-H1.3x^−^ cell lines when compared to parental HepG2 cells (Figure 3a). HepG2 cells express a stabilized mutant β-catenin, S33A, and thus are already more active [17, 24], suggesting perhaps that HBV simply enhanced this activity. To furthermore exclude a clonal cell selection we also repeated the analysis in Huh7 cells, which express wildtype β-catenin, to evaluate whether HBV is also initiating β-catenin-signalling. After transient transfection with H1.3 and H1.3x^−^ expressing plasmids, HBV replication in Huh7 cells was confirmed by measuring progeny virus release (Figure 3b). A 9-fold increase in Tcf/LEF reporter activity was found in both transiently transfected Huh7 cell lines (Figure 3b). Importantly, HBV induced the expression of the β-catenin target gene c-myc (Figure 3c), further confirming active β-catenin-signalling in these cells. These results demonstrate that HBV initiates and enhances β-catenin-signalling in an HBx independent fashion.

### HBV replication and β-catenin-signalling are regulated by Src kinase activity

**Figure 4:**
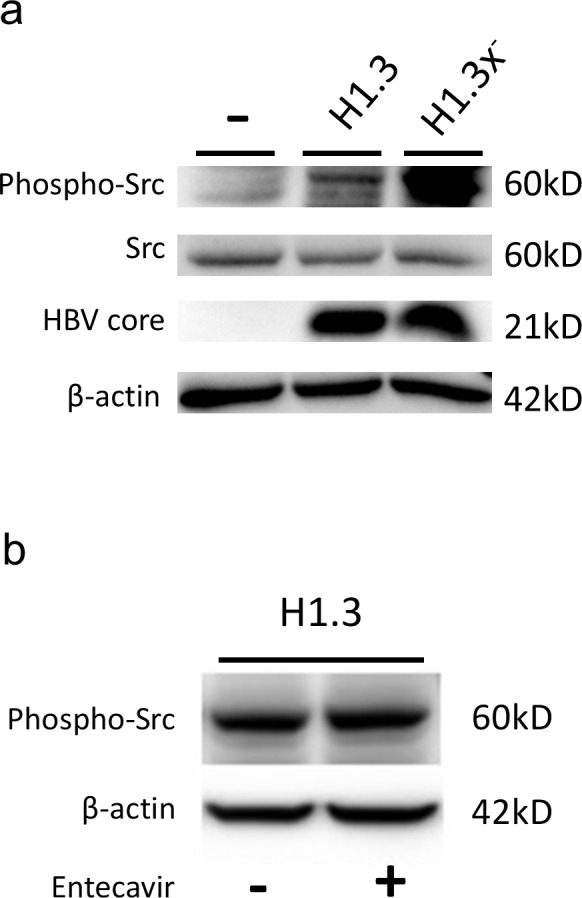
c-Src kinase activity in HBV-replicating hepatoma cells **(a)** Total protein levels of c-Src kinase and its tyrosine 416 phosphorylated activated form (Tyr416) as well as HBV core protein were subjected to Western blot analysis in parental (−) compared to HBV-replicating (H1.3 + H1.3x^−^) HepG2 cells. **(b)** Total protein levels of the c-Src tyrosine 416 phosphorylated activated form (Tyr416) were subjected to Western blot analysis in HBV-replicating HepG2H1.3 cells either treated with the reverse transcriptase inhibitor Entecavir (+) or not (−).

To dissect the mechanism by which HBV leads to increased β-catenin-signalling we analyzed c-Src kinase activity, which has been shown to promote reverse transcription and DNA replication in HBV infection [4]. Western blot analysis showed a pronounced increase of activated, phospo-Tyr416-Src levels in HBV-replicating HepG2 as compared to control cells (Figure 4a). Next, we investigated whether HBV replication itself leads to an upregulation of phospo-Tyr416-Src levels. Blocking HBV replication by the reverse transcriptase inhibitor Entecavir in HepG2H1.3 cells (Figure 4b) or in HepG2.2.15 cells (data not sown) did neither show an effect on total nor on phospo-Tyr416-Src levels.

**Figure 5:**
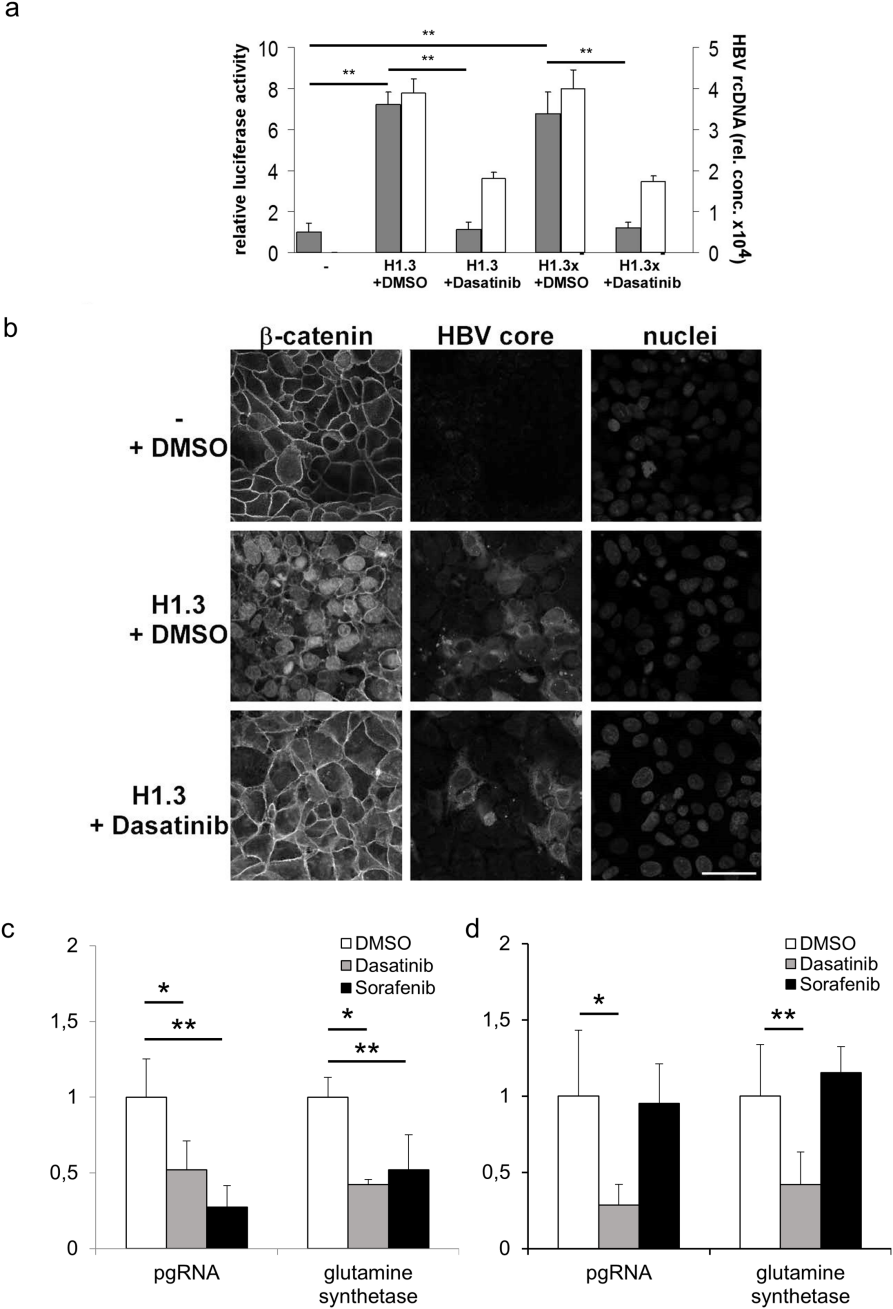
Tcf/LEF dependent β-catenin-signalling and target gene expression in hepatoma cells **(a)** Relative luciferase activity indicates the level of Tcf/LEF dependent β-catenin-signalling (grey bars) in parental (−), H1.3x and H1.3x^−^ transfected Huh7 cells either treated with Dasatinib or DMSO. The ratio in parental cells was set to 1. Active viral replication was determined by measuring HBV rcDNA (white bars) levels in supernatants of HBV-producing cells. The assays were performed in independent triplicates, means + SD are shown. ^**^ p<0.01 vs parental cells and HBV replicating cells respectively, t-test. **(b)** Immunofluorescence staining of β-catenin in parental (−) and HBV-producing H1.3 Huh7 cells either treated with Dasatinib or DMSO. Co-staining of HBV core protein and nuclei. Scale bar indicates 40μm. pgRNA and β-catenin-signalling dependent target gene mRNA were quantified by real-time PCR in **(c)** HBV infected HepG2-NTCP and **(d)** HBV infected Huh7.5-NTCP cells treated either with Dasatinib (grey bars) or Sorafenib (black bars) relative to DMSO treated HBV infected cells (white bars, ratios in DMSO treated cells were set to 1). Normalized expression ratios were relative to housekeeping gene GAPDH. The assays were performed in independent sixplicates, means + SD are shown. ^*^ p< 0.05; ^**^ p<0.01 vs DMSO treated HBV infected cells, t-test.

To examine if increased c-Src kinase activity is indeed involved in HBV replication, activation of β-catenin-signalling and disassembly of adherens junctions, we treated HBV-replicating Huh7 cells with Dasatinib. This ATP-competitive tyrosine kinase inhibitor inhibits all members of Src family, including c-Src, Lck, Fyn and Yes [40, 41]. Dasatinib treatment reduced Tcf/LEF dependent β-catenin-signalling in Huh7 cells replicating HBV (H1.3) or x-deficient HBV (H1.3x^−^) to levels observed in parental control cells (Figure 5a, grey bars). In addition, Dasatinib reduced HBV gene expression 7- to 8-fold as demonstrated by progeny virus release (Figure 5a, white bars) and HBeAg ELISA (data not shown). Moreover, immunofluorescence analysis of HBV-replicating Huh7 cells treated with Dasatinib showed that inhibition of Src phosphorylation also prevented the disassembly of adherens junctions induced by HBV (Figure 5b).

Since Dasatinib treatment reduced Tcf/LEF dependent β-catenin-signalling in HBV transfected cells, we next investigated the effects of Dasatinib treatment on HBV replication and β-catenin target gene mRNA glutamine synthetase (GS) expression levels in HBV infected HepG2-NTCP and Huh7.5-NTCP cells, which are permissive to HBV and allow investigating the full viral life cycle [42]. In addition, we compared the effects of Dasatinib treatment to that of Sorafenib, a tyrosine kinase inhibitor that inhibits Raf kinase and the vascular endothelial growth factor receptor intracellular kinase pathway [43] and is the recommended treatment for advanced HCC (Grade 1B) [44]. In HBV infected HepG2-NTCP cells that carry S33A mutated β-catenin, HBV pgRNA as well as GS expression levels were significantly reduced to the same extend by both drugs (Figure 5c). In HBV infected Huh7.5-NTCP cells that carry wildtype β-catenin in contrast, HBV pgRNA as well as GS expression levels were reduced by Dasatinib but not by Sorafenib (Figure 5d). These results strongly indicate a role of Src kinase not only in HBV infection but also in HBV induced β-catenin translocation and signalling that maybe therapeutically targeted by tyrosine-kinase inhibitor treatment.

## DISCUSSION

In this study we identify a link between HBV, increased Src kinase activity, adherens junction instability and enhanced β-catenin-signalling. We show that HBV induces phosphorylation of c-Src kinase leading to activation of HBV transcription and replication as well as β-catenin-signalling and disassembly of adherens junctions independent of the viral transactivator HBx. These processes can be modified by the tyrosine kinase inhibitors Dasatinib and Sorafenib. Our results suggest that increased β-catenin-signalling in the liver is one route by which HBV contributes to HCC development.

A previous report showed that HBx overexpression leads to a downregulation of E-cadherin in cell culture [45]. In our study, E-cadherin protein levels did not correlate with HBV replication - neither in hepatoma cells nor in liver tissue of HBV-infected patients. In chronically HBV-infected patients, E-cadherin levels were rather heterogeneous and showed a high inter-individual variation - not only in tumor but also in non-tumor tissue. Nevertheless, even though overall levels were not affected by HBV, we did observe a strong effect of HBV replication on E-cadherin localization with reduced cell surface expression and a more punctate cytoplasmic localization, suggesting increased internalization of E-cadherin. Alternatively, HBV may also reduce efficient transport to the cell surface. A previous study showed that phosphorylation of the cytoplasmic domain of E-cadherin by Src results in increased internalization into endosomes, which are then either degraded in the lysosome or recycled back to the cell surface [46]. Src also phosphorylates β-catenin at specific tyrosine residues, particularly at Y142 and Y654, which reduces its affinity for α-catenin and E-cadherin, respectively [47, 48], thus promoting disassembly of the cadherin complex. As overexpression of E-cadherin sequesters β-catenin thus reducing its nuclear signalling activity [49–51], HBV induced disassembly of the junctions through Src may induce stabilization of β-catenin [52], and subsequent increased transcriptional activity. Moreover, HBV dependent activation of Src also suppresses glycogen synthase kinase 3 (GSK3) activity, the central kinase that targets β-catenin for proteosomal degradation, hence more directly stabilizing β-catenin.

In our experiments, HBV increased c-Src activity in HepG2 cells and specific inhibition of Src activity strongly reduced viral pgRNA transcription and replication, showing a bidirectional activation. This is in line with previous studies reporting that Src promotes reverse transcription as well as HBV-DNA replication [4, 5, 53]. However, in contrast to what was suggested previously [5], c-Src activation did not require HBx.

Previous studies showed that overexpression of HBx either disrupts adherens junctions [16] or induces epithelial-mesenchymal transition [54, 55] through Src activation or increases Wnt-dependent β-catenin-signalling [17, 56, 57]. Although overexpression of HBx is apparently able to induce these effects, in our study expression of HBx at moderate levels under its endogenous promoter did not do so. Importantly, deletion of HBx also did not affect the ability of HBV to induce c-Src, translocate E-cadherin or increase β-catenin-signalling. HBV (even without HBx) was sufficient to enhance constitutive active β-catenin-signalling in HepG2 cells carrying a mutant β-catenin and, more importantly, to induce β-catenin-signalling in Huh7 cells that are wildtype for β-catenin.

Over the last years there has been an increasing interest in identifying new compounds that modify signalling pathways in HCC. It has been shown that antagonists of Tcf4/beta-catenin complex inhibit the growth of HCC cells *in vitro* and *in vivo* [58] highlighting the important role of the Wnt/β-catenin-signalling in HCC [59, 60]. In addition, overexpression of Src in association with reduced expression of a negative regulator of Src, the C-terminal Src kinase, has been observed [61, 62] and activation of the Src family of tyrosine kinases has been linked to adherens junction disassembly, increased β-catenin-signalling and cancer progression [63, 64].

These data as well as the results from our study indicate that targeting Src kinase might be a promising and specific therapeutic strategy against HBV-associated HCC. So far, only the multi-kinase inhibitor Sorafenib is approved for treatment of advanced HCC. In our study Sorafenib and Dasatinib were comparably effective in suppressing HBV replication and the expression of Tcf/LEF dependent β-catenin-signalling target gene GS in HBV-infected HepG2-NTCP cells, that express a mutant, stabilized β-catenin. Interestingly, in HBV infected Huh7.5-NTCP cells, which express wildtype β-catenin, and therefore represent an important subgroup of HCCs, only Dasatinib treatment showed an effect. Thus, Dasatinib, which has already received clinical approval by American and European authorities for treatment of leukemia, may be an interesting treatment option for HBV-dependent HCC.

Taken together, we have shown that HBV induces Src kinase dependent β-catenin-signalling and disassembly of adherens junctions, which interestingly does not require the viral transactivator HBx. Importantly, these HBV-dependent processes can be modified by the tyrosine kinase inhibitor Dasatinib. Our results provide new insights into HBV-host cell interaction and help to understand the molecular basis of HBV-associated carcinogenesis while also providing potentially novel therapeutic approaches against HBV.

## MATERIALS AND METHODS

### Cell culture, transfection/infection, reporter assay and drugs

Hepatoma cell lines HepG2, Huh7 and Huh7.5 were cultivated as described [38]. HepG2-H1.3 (clone2) and HepG2-H1.3x^−^ (clone6) cells were generated by stable transfection with plasmids pTH1.3x^+/−^ (Supplementary Table 1). Cells were plated onto collagen-coated dishes and at 80-90% confluency maintained in WilliamsE/DMEM 1:1 containing 1% fetal calf serum [38, 65].

Huh7 cells were transiently transfected with pTH1.3x^+/−^ using Fugene 6 Transfection Reagent (Roche Diagnostics GmbH, Mannheim, Germany).

HepG2-NTCP and Huh7.5-NTCP cells were cultivated and infected with HBV as described [66]. The multiplicity of infection (MOI) was 150 enveloped, DNA-containing HBV virions per cell expected to result in a 30-40% infection rate in HepG2 cells.

To determine β-catenin-signalling activity, reporter plasmids were cotransfected (Supplementary Table 1). Three days after transfection, luciferase signals were measured and relative luciferase activity was determined as described [67, 68].

Dasatinib (Bristol-Myers Squibb, New York, NY, USA) and Sorafenib (LC Laboratories, Woburn, MA, USA) were diluted in DMSO and used for cell treatment (concentrations 2, 5 μg ml-1 and 1, 25 μg ml-1 respectively). Drug treatment was started one day post infection with HBV. Medium and drug substances were changed every two days.

Entecavir (Bristol-Myers Squibb, Uxbridge, UK) was reconstituted in water and used for cell treatment at a final concentration of 5 μM. Drug treatment was started 1 day after cell seeding when they reached 95-100% confluence. Medium and drug substances were changed every 3 days. Cells were harvested at day 7.

Hepatitis B e antigen (HBeAg) was determined by a commercial assay (Axsym™, Abbott Diagnostics, Wiesbaden, Germany). The HBeAg concentration in ng/ml was calculated using an International Standard for Hepatitis B Virus e Antigen from the Paul-Ehrlich-Institut.

### Human liver tissue

Snap frozen human HCC and non-tumor (peritumor) HBV-infected liver tissue samples (pat1-pat4) were selected from the tissue bank of the Institute of Pathology, University Hospital Cologne, established after informed consent of patients. Selection criteria were: active HBV infection (seropositivity for HBsAg and anti-HBc and/or detectable HBV viremia), absence of other obvious cause for HCC (e.g. hepatitis C virus (HCV) infection, hematochromatosis), absence of human immunodeficiency virus (HIV) infection, and no serological evidence for acute infection with or reactivation of cytomegalovirus (CMV) or Epstein-Barr virus (EBV) infection. Tumors were graded according to the American Joint Commission on Cancer (Table 1). Non-tumor/HBV-negative liver tissue samples were obtained from human liver grafts (HBV, HCV, HIV negative) not suited for transplantation (pat5-pat8). The use of human tissue was approved by the local ethics committee and was in accordance with ethical standards as formulated in the Helsinki declaration.

**Table 1:** Staging and grading of human hepatocellular carcinoma samples

Patient number	Serum HBsAg/antiHBc	Tumor staging and grading	Peritumor, fibrosis stage	Peritumor inflammation grade
1	+	pT3 N0 Mx G2 R0	3	3
2	+	pT3 N0 Mx G3 R0	4	1
3	+	pT1 N0 Mx G2 R0	2-3	2
4	+	pT1 N0 Mx G1 R0	4	2

### Real-time PCR

One μg of total RNA extracted from HepG2 and Huh7.5 cells using total RNA extraction kit (Qiagen, Hilden, Germany) was transcribed into cDNA after DNase digestion using SuperScript II Reverse Transcriptase (Invitrogen, Carlsbad, USA). HBV pgRNA was detected as described [69]. For gene expression analysis, appropriate exon-exon spanning primer pairs were selected (Supplementary Table 2). To distinguish HBV-genomic-DNA from HBV-plasmid-DNA used for transfections, rcDNA specific primers HBV3054fw 5′-ACTAGGAGGCTGTAGGCATA-3′, HBV132rev 5′-AGACTCTAAGGCTTCCCG-3′ were designed. Real-time PCRs were performed using the LightCycler™ system and normalized to a dilution series of calibrator cDNA using the Relative Quantification Software (both Roche Diagnostics) as described [69].

### Protein expression analysis

Total proteins from cells and liver tissue were isolated using SDS lysis buffer (15 mM Tris/HCl, pH 6.8, 2.5% glycerol, 0.5% SDS, 1mM EDTA with protease complete cocktail). Equal amounts of protein (10-20 μg) were separated by 7.5 to 12.5 % SDS-PAGE, transferred onto nitrocellulose membranes, stained with appropriate primary (Supplementary Table 3) and secondary antibodies (Sigma, Deisenhofen, Germany) and visualized by ECL Western Blot Detection Reagent (Amersham Bioscience, Buckinghamshire, England).

Cell surface proteins were biotinylated using a biotinylation kit (Pierce, Rockford, IL, USA), precipitated using streptavidin coupled agarose beads and analyzed by Western blot.

For immunofluorescence analysis, cells were fixed with 100% ice-cold methanol and stained with appropriate primary (Supplementary Table 3) and secondary antibodies (Alexa Fluor™ 488 or 594 conjugate (Molecular Probes, Eugene, OR, USA)). Nuclei were stained with Diamino-2-phenylindol (DAPI). Fluorescence images were acquired using confocal microscope FluoView1000 (Olympus, Hamburg, Germany).

### Statistical analysis

All data were expressed as means ± SD (for number of replicates see figure legends). Data were tested for normality as well as equal variance and analyzed using a two-tailed t test for equal or unequal variance, as appropriate. p values <0.05 were considered statistically significant.

## SUPPLEMENTARY MATERIALS TABLES



## Abbreviations

HBV: hepatitis B virus
HCC: hepatocellular carcinoma
HBx: HBV X protein
EMT: mesenchymal transition
HBeAg: hepatitis B e antigen
pgRNA: pregenomic RNA
ALAS: aminolevulinate synthase
GS: glutamine synthetase
NTCP: sodium taurocholate co-transporting polypeptide
