# Supplementary material for: Hepatitis B virus promotes β-catenin-signalling and disassembly of adherens junctions in a Src kinase dependent fashion
Source: Oncotarget. 2018 Sep 21;9(74):33947–60. doi: 10.18632/oncotarget.26103 (PMC6188061; doi:10.18632/oncotarget.26103)
Supplement: Supplementary file 1 [file oncotarget-09-33947-s001.pdf]

Hepatitis B virus promotes β-catenin-signalling and disassembly of adherens junctions in a Src kinase dependent fashion

SUPPLEMENTARY MATERIALS

Supplementary Table 1: Plasmids used for transfection of cells in 6 well plates (7.8 x 10^5 cells per well)

Plasmid (Concentration)	Construct	Manufacturer/Provided by
pTH1.3 (2 µg/well)<br>pTH1.3x$^-$ (2 µg/well)	1.3-fold overlength HBV genome [1]<br>1.3-fold overlength HBV genome + premature stop-codon into 5' and 3' X ORF confirmed by sequencing [1]	Heinz Schaller, (Center for Molecular Biology, Heidelberg, Germany)
pGL3/TOPflash (150 ng/well)<br>pGL3/FOPflash (150 ng/well)	SV40 promoter containing *firefly* luciferase gene under control of 5x consensus<br>wild-type Tcf/LEF binding elements or mutant Tcf/LEF binding elements [2]	Hans Clevers (Hubrecht Laboratory, Utrecht, The Netherlands)
pRL-tk (37.5 ng/well)	Herpes simplex virus thymidine kinase promoter containing *renilla luciferase* as internal control reporter	Promega (Mannheim, Germany)

Supplementary Table 2: Sequences of primers used for LightCycler™ real-time PCR

Target gene	Sequence forward	Sequence reverse
E-cadherin	CCCCAGAGGATGACAC	GGGTCAGTATCAGCCG
β-catenin	AACGGCTTTCAGTTGAG	TTGCTGTCACCTGGAG
aminolevulinate synthase (ALAS)	CAACATCTCAGGCACCAGTA	CTCCACTGTTACGGATACCT
GAPDH	GGTATCGTGGAAGGACT	GGGTGTCGCTGTTGAA
glutamine synthetase (GS)	AAGTGTGTGGAAGAGTTGCC	TGCTCACCATGTCCATTATC

Supplementary Table 3: Primary antibodies used for Western blot (WB) and immnuofluorescence (IF)

Target	Source	Working dilution	Manufacturer/Provided by
β-catenin (monoclonal antibody, (mAb))<br>E-cadherin (mAb)	Mouse<br>Mouse	1:500 (WB + IF)<br>1:2500 (WB)	BD Transduction Laboratories (San Jose, CA, USA)
Human-E-cadherin (mAb)	Mouse	1:100 (WB)	Clone HECD1, Zymed Laboratories Inc. (San Francisco, CA, USA)
Src (mAb)<br>Phospho-src family (Tyr416) (polyclonalAb)	Rabbit<br>Rabbit	1:1000 (WB)<br>1:1000 (WB)	Cell Signaling (Danvers, MA, USA)
c-myc	Mouse	2μg/ml (WB)	BD Biosciences Pharmingen (San Jose, CA, USA)
HBV core (H800) (polyclonalAb)	Rabbit	1:10000 (WB)<br>1:5000 (IF)	Heinz Schaller (Center for Molecular Biology, Heidelberg, Germany).

REFERENCES

1. Untergasser A, Protzer U. Hepatitis B virus-based vectors allow the elimination of viral gene expression and the insertion of foreign promoters. Hum Gene Ther. 2004; 15:203–10.

2. Korinek V, Barker N, Morin PJ, van Wichen D, de Weger R, Kinzler KW, Vogelstein B, Clevers H. Constitutive transcriptional activation by a beta-catenin-Tcf complex in APC-/- colon carcinoma. Science. 1997; 275:1784–87.
